# Construction and Analysis of lncRNA-Associated ceRNA Network in Atherosclerotic Plaque Formation

**DOI:** 10.1155/2022/4895611

**Published:** 2022-04-15

**Authors:** Danyu Li, Yunci Ma, Weiqian Deng, Jianhua Feng

**Affiliations:** ^1^Guangzhou Xinhua University, 248 Yanjiangxi Road, Machong Town, Dongguan, Guangdong 523133, China; ^2^The Fifth Affiliated Hospital, Southern Medical University, No. 566, Congcheng Avenue, Conghua District, Guangzhou, Guangdong 510900, China

## Abstract

Atherosclerosis (AS) is a vascular disease with plaque formation. Unstable plaques can be expected to result in cardiovascular disease, such as myocardial infarction and stroke. Studies have verified that long noncoding RNAs (lncRNAs) play a critical role in atherosclerotic plaque formation (APF), including MALAT1, GAS5, and H19. A ceRNA network is a combination of these two interacting processes, which regulate the occurrence and progression of many diseases. However, lncRNA-associated ceRNA network in terms of APF is limited. This study sought to discover novel potential biomarkers and ceRNA network for APF. We designed a triple network based on the lncRNA-miRNA and mRNA-miRNA pairs obtained from lncRNASNP and starBase. Differentially expressed genes (DEGs) and lncRNAs in human vascular tissues derived from the Gene Expression Omnibus database (GSE43292, GSE97210) were systematically selected and analyzed. A ceRNA network was constructed by hypergeometric test, including 8 lncRNAs, 243 miRNAs, and 8 mRNAs. APF-related ceRNA structure was discovered for the first time by combining network analysis and statistical validation. Topological analysis determined the key lncRNAs with the highest centroid. GO and KEGG enrichment analysis indicated that the ceRNA network was primarily enriched in “regulation of platelet-derived growth factor receptor signaling pathway,” “negative regulation of leukocyte chemotaxis,” and “axonal fasciculation.” A functional lncRNA, HAND2-AS1, was identified in the ceRNA network, and the main miRNA (miRNA-570-3p) regulated by HAND2-AS1 was further screened. This present study elucidated the important function of lncRNA in the origination and progression of APF and indicated the potential use of these hub nodes as diagnostic biomarkers and therapeutic targets.

## 1. Introduction

Atherosclerotic cardiovascular disease is a major threat to human health and quality of life in modern society [1]. The formation of atherosclerotic plaques is one of the major causes of cardiovascular disease morbidity and mortality, such as myocardial infarction and stroke [2]. However, the underlying mechanism of atherosclerotic plaque formation (APF) is not entirely clear, so further exploration of the possible effective treatments has drawn substantial attention.

Noncoding RNAs (ncRNAs) refer to the functional RNAs that cannot encode proteins in transcriptome, including microRNAs (miRNAs) and long noncoding RNAs (lncRNAs). miRNAs are a class of highly conserved single-stranded noncoding small RNAs with posttranscriptional regulatory activity. Long noncoding RNAs (lncRNAs) are longer than 200 nucleotides with no protein coding capacity or limited protein coding potential [3–7]. Although ncRNAs cannot code proteins, they can exert their functions by regulating the coding RNAs. Studies have shown that functional ncRNA not only play an important role in cell proliferation, differentiation, and aging but also participate in the occurrence and development of many diseases, such as cancer, digestive system diseases, and cardiovascular diseases [8–13]. Atherosclerosis (AS) is a vascular disease with plaque formation. Unstable plaques can be expected to result in cardiovascular disease, such as myocardial infarction and stroke. Studies have verified that long noncoding RNAs (lncRNAs) play a crucial role in APF, including MALAT1, GAS5, and H19 [14–16].

**Figure 1:**
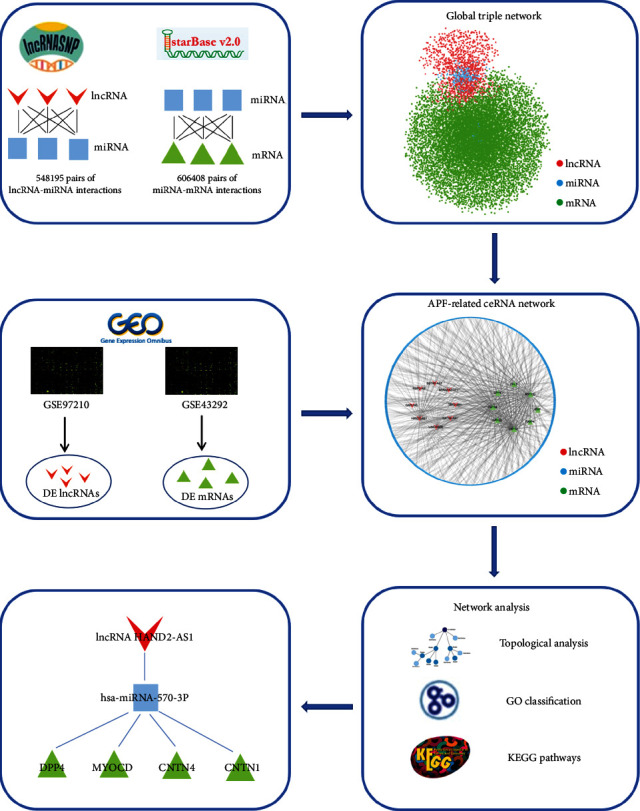
Flowchart for an overview of the present analysis.

Competitive endogenous RNA (ceRNA) is the basis of a novel regulatory mechanism between noncoding RNA and coding RNA. lncRNA can be used as ceRNA to competitively bind miRNA with target genes, thereby reducing the inhibitory effect of miRNAs on target genes [17]. Therefore, they can indirectly regulate the expression of target genes and thus participate in the development of diseases. For instance, Wang et al. found that long noncoding RNA SNHG1 regulates NOB1 expression by sponging miR-326 and promotes tumorigenesis in osteosarcoma [18]. Li et al. discovered that the long noncoding RNA CRNDE acts as a ceRNA and promotes glioma malignancy by preventing miR-136-5p-mediated downregulation of Bcl-2 and Wnt2 [19]. However, the mechanism of ceRNA associated with APF is still unclear. Therefore, this study was aimed at investigating the functional role of novel potential biomarkers associated with lncRNA in the progression of APF by lncRNA-associated ceRNA network analysis. The flow chart of this research is shown in Figure 1.

## 2. Materials and Methods

### 2.1. Construction of the Global Triple Network

In our study, lncRNA-miRNA interactions and miRNA-mRNA interactions were obtained from the lncRNASNP database (http://bioinfo.life.hust.edu.cn/lncRNASNP/) [20] and starBase V2.0 database (http://starbase.sysu.edu.cn/) [21], respectively. Then, we constructed the global triple network and visualized by Cytoscape software v3.9.0 (http://www.cytoscape.org/). Cytoscape is a popular bioinformatics package for biological network visualization and data integration [22]. We defined the lncRNA, miRNA, and mRNA as the nodes of the network in Cytoscape software. If they could interact with each other, we connected them with lines. Finally, a global triple ceRNA network was constructed.

### 2.2. Screening of Differentially Expressed Genes (DEGs)

To identify the main DEGs between normal blood vessels and the blood vessels containing atherosclerotic plaques, microarray data GSE97210 and GSE43292 were downloaded from the GEO database (https://www.ncbi.nlm.nih.gov/gds/). When we enter the keywords “lncRNA” and “atherosclerotic plaque” in the GEO dataset search box, the result shows that there is only the GSE97210 dataset. The GSE97210 lncRNA dataset was measured using GPL16956 Platforms (Agilent-045997 Arraystar human lncRNA microarray V3) and included data from 3 patients with APF and 3 healthy controls. The mRNA expression data in GSE43292 were from 32 patients with APF and 32 healthy controls and were based on GPL6244 Platforms (Affymetrix Human Gene 1.0 ST Array). Subsequently, data were imported into the R-studio and normalized with RMA algorithm [23]. DEGs were defined by the Bioconductor/R limma package as previously described [24]. Selected values of *P* < 0.05 and [logFC] > 1.5 were applied.

Since the differentially expressed lncRNAs that have been screened out were replaced by sequence numbers of probes, they were further reannotated through the BLAST software (https://blast.ncbi.nlm.nih.gov/Blast.cgi).

### 2.3. Detecting Potential ceRNA Mechanism

A hypergeometric distribution is a discrete probability distribution in statistics. It was employed to detect the competing lncRNA-mRNA interactions and evaluated the significance of the same miRNA between each mRNA and lncRNA. In this study, we used this statistical method to detect potential ceRNA mechanism, which made the results more objective and scientific. The *P* value was calculated by the following equation:

(1) $$\begin{array}{c} P=1-\overset{X}{\underset{t=0}{\sum }}\;\frac{\left(\begin{array}{c} M\\ t \end{array}\right)\left(\begin{array}{c} K-M\\ N-t \end{array}\right)}{\left(\begin{array}{c} K\\ N \end{array}\right)}. \end{array}$$

where *K* represented the count of human genome miRNA, *M* represented the count of miRNA related to the mRNA, *N* represented the count of miRNA related to the lncRNA, and *X* represented the count of miRNA shared between lncRNA and mRNA.

Then, the original *P* values were corrected for false discovery rate (FDR) according to the Benjamini and Hochberg method [25]. We considered that FDR < 0.01 was statistically significant.

### 2.4. GO Enrichment and Signaling Pathway Analysis

DEGs were subjected to gene ontology (GO) enrichment using Cytoscape plugin ClueGO to explore their roles in various biological processes. After GO functional enrichment analysis, we considered biology process terms with *P* value < 0.05 to be statistically significant. With the KEGG option, the signaling pathways regulated by disease-related genes could be obtained. ClueGO network was constructed by using kappa statistics and showed the relationship between terms with similarly associated genes. These specific and common terms were automatically calculated [26].

### 2.5. Construction of ceRNA Network Related to APF

The differentially expressed lncRNAs and mRNAs were marked from the global triple network and found out the lncRNA-miRNA interactions and mRNA-miRNA interactions, respectively. Then, these interactions were combined by hypergeometric test and visualized through Cytoscape software. A new lncRNA-miRNA-mRNA network related to APF was constructed.

### 2.6. Definition of Topological Features for the ceRNA Network

Topological parameters mainly include degree, closeness, and betweenness centrality. These are the key criteria for measuring the importance of network nodes. The NetworkAnalyzer tool in Cytoscape was used to calculate and analyze the topological parameters of each node in the network, including degree, closeness, and betweenness centrality. The most commonly used and the most straightforward measurement of centrality in network analysis is the degree value. As reported, degree is defined as the quantity of links that a node connects with other nodes [27]. Closeness is defined as the average mean path from a node to all other nodes reachable from it [28]. Betweenness is an index evaluating the influence a node exerts over the spread of information through the network [29]. Finally, we screened the lncRNA with the highest value of 3 standards in the network; that is to say, this lncRNA is the major lncRNA that regulates APF.

It was found that the selected lncRNA and its downstream miRNAs and target genes constituted a new network from the global triple ceRNA network. lncRNAs with the highest degree were screened out of the new network. The major miRNA and its target genes regulated by lncRNA were constructed into a new small ceRNA network. Finally, functional analysis of the small regulatory network was performed to verify whether it was involved in the regulation of the APF-related ceRNA network.

## 3. Results

### 3.1. Construction of a Global Triple ceRNA Network

**Figure 2:**
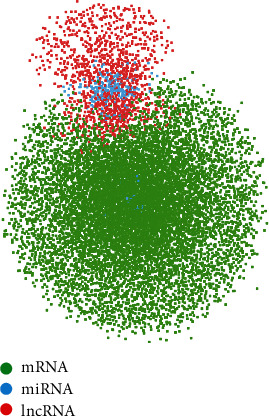
The global triple ceRNA network. The green nodes represent mRNAs, the red nodes represent lncRNAs, and the blue nodes represent miRNAs.

In order to construct a global triple network, 548195 lncRNA-miRNA interaction pairs were downloaded from the lncRNASNP database, and then, 606408 miRNA-mRNA interaction pairs were downloaded from the starBase V2.0 database. Then, we integrated all miRNA-mRNA interaction pairs and lncRNA-miRNA interaction pairs into a global triple ceRNA network through the Cytoscape software. Afterwards, we defined lncRNAs, miRNAs, and mRNAs as the nodes of the network. If they could interact with each other, we connected them with lines, completing a global triple ceRNA network (Figure 2).

### 3.2. Screening of Genes Related to APF

**Figure 3:**
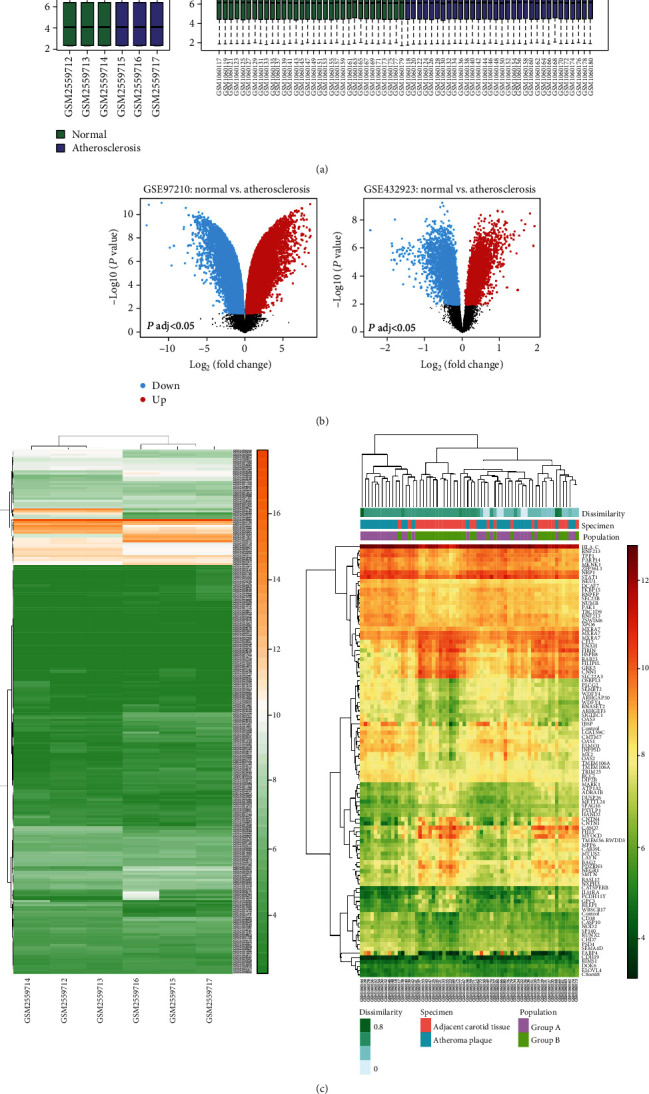
Identification of differentially expressed genes from the GSE97210 dataset and the GSE43292 dataset. (a) The boxplots of the sample expression level in GSE97210 and GSE43292. (b) The volcano plot of the differential expressed genes between the APF and control group in GSE97210 and GSE43292. (c) Heat map of the expression profiles including DEGs and lncRNAs between the APF and control group in GSE97210 and GSE43292.

A total of two gene expression profiles (GSE97210, GSE43292) were downloaded from the GEO database, comparing the blood vessels between patients with APF and healthy controls. 39 differentially expressed lncRNAs (DELs) and 9 differentially expressed mRNAs (DEMs) were identified with [logFC] > 1.5 and adjusted *P* < 0.01 (Tables 1 and 2), including 30 genes upregulated and 18 genes downregulated in the APF group. Since these lncRNAs screened out are represented by the sequence of the probe, they are further reannotated by BLAST software. These overview boxplot, volcano plot, and heat map of the DEGs were obtained by GEO2R and R-studio (Figure 3).

### 3.3. Construction of the Network Related to APF from Global Triple ceRNA Network

**Figure 4:**
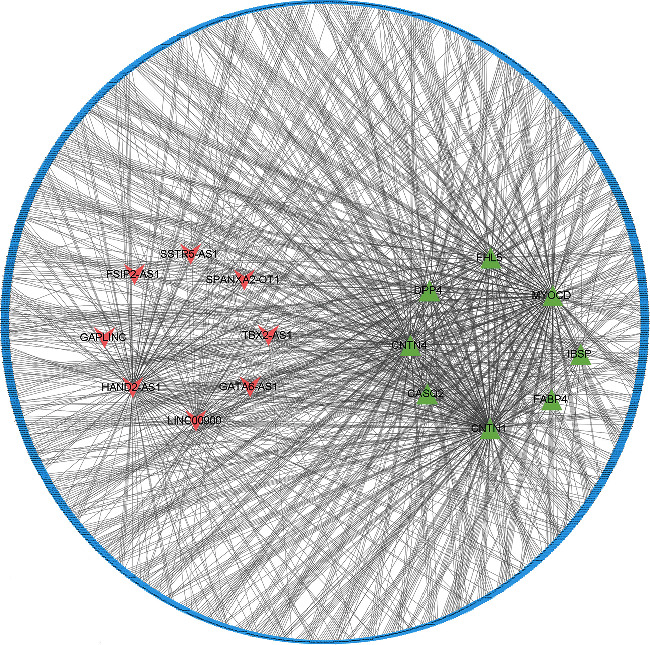
Construction of the network related to APF by Cytoscape. The green nodes represent mRNAs, the red nodes represent lncRNAs, and the blue nodes represent miRNAs.

First, the lncRNASNP database was used to predict differentially expressed miRNAs (DEMis) associated with DELs. The starBase V2.0 database was used to predict miRNAs that interact with DEGs. Then, a hypergeometric test was utilized to further detect the significance of these lncRNA-miRNA pairs and mRNA-miRNA pairs. Finally, we constructed a new lncRNA-miRNA-mRNA network related to APF in Cytoscape software (Figure 4). It consists of 8 lncRNA nodes, 243miRNA nodes, 8 mRNA nodes, and 1298 edges.

### 3.4. Functional Enrichment Analyses of APF-Related Network

**Figure 5:**
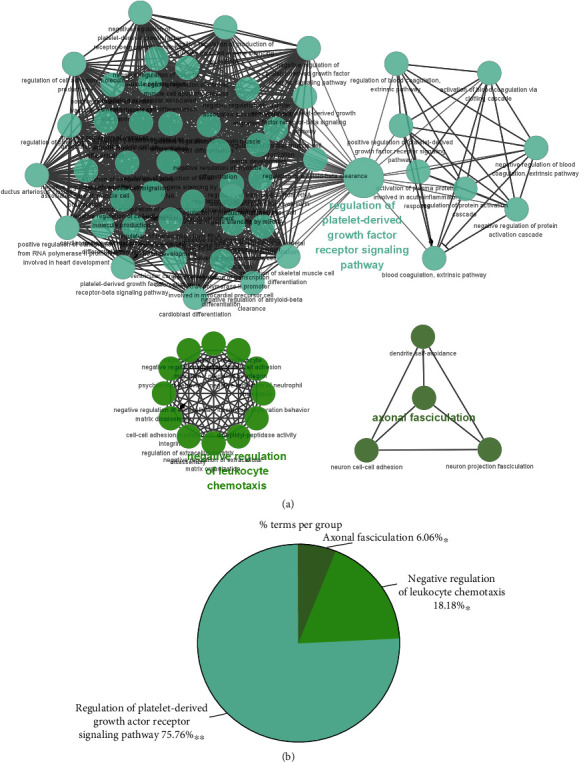
Signaling pathway enrichment analysis for APF-related genes by Cytoscape. (a) A functionally grouped network of enriched categories for target genes. Every node is a disease-related GO term. (b) The proportion of each term.

Enrichment analysis of GO classification and KEGG pathway was performed on the DEGs in APF-related networks by employing ClueGO plugin in Cytoscape software. The results are shown in Figure 5. GO enrichment analysis has shown that 66 GO terms were enriched. KEGG pathway suggested that APF-related genes were mainly related with the “regulation of platelet-derived growth factor receptor signaling pathway.”

### 3.5. Topological Analysis of ceRNA Network Related to APF

**Figure 6:**
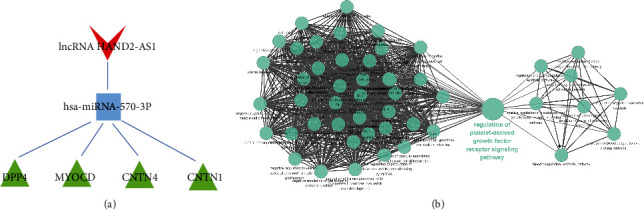
ceRNA regulatory network of the miRNA bound by HAND2-AS1 and the gene function of the network. (a) The ceRNA network of hsa-miRNA-570-3p sponged by lncRNA HAND2-AS1. (b) Gene function analysis of the ceRNA network.

First, we used Cytoscape software to calculate the topology parameters of the nodes in the APF-related ceRNA network. Then, all of the nodes' topological parameters in the network were ranked (Table 3). It was previously reported that the degree value of a hub node should not be less than 5 [30]. A comprehensive examination revealed that three parameters of lncRNA HAND2-AS1 were significantly higher than those of other lncRNA nodes. Therefore, HAND2-AS1 was the hub node of this ceRNA network. In other words, HAND2-AS1 may be the key lncRNA regulating the development of APF. Further, we extracted miRNAs and mRNAs associated with the HAND2-AS1 from the APF-related ceRNA network and constructed a new ceRNA network of HAND2-AS1 by Cytoscape software (Figure 6(a)). Among the top 3 miRNAs (hsa-miRNA-466, hsa-miRNA-570-3p, and hsa-miRNA-4495), only hsa-miRNA-570-3p could directly interact with HAND2-AS1. The mRNAs that directly interact with hsa-miRNA-570-3p are CNTNI, MYOCD, CNTN4, and DPP4. KEGG functional enrichment analysis indicated that hsa-miRNA-570-3p may also act on the “regulation of platelet-derived growth factor receptor signaling pathway” (Figure 6(b)).

## 4. Discussion

APF is a complex disease that has brought a heavy burden to people all over the world. Since the pathogenesis and process of this disorder are complex, it is necessary to solve the problem through a more comprehensive perspective. In recent years, with the rapid development of high-throughput genomics, noncoding RNA has received extensive attention. Noncoding RNA includes lncRNA and miRNA, which are involved in the regulation of multiple biological processes [31]. ceRNA is a common regulatory mechanism between lncRNA and miRNA, and it is an important method in bioinformatics analysis [32, 33]. However, the APF-related ceRNA regulation mechanism has not been systematically elucidated. In the current research, a synthetical bioinformatics method was applied to identify the key lncRNA involved in APF and further explore the molecular mechanism of APF.

First of all, construct a global triple network based on the ceRNA theory by using the interactive data of lncRNASNP and starBase [34]. Then, we collected the DEGs associated with APF by mining microarray data from the GEO database. A ceRNA network related to APF was extracted by mapping the DEGs into the triple global network. It contains 8 mRNA nodes, 243 miRNA nodes, 8 lncRNA nodes, and 1298 edges. Further, we applied the topological features and cluster analysis to the APF-related ceRNA network. Finally, with comprehensive consideration of degree, closeness, and betweenness centrality, HAND2-AS1 was considered to be a possible hub node. In other words, these results suggest that HAND2-AS1 might play an important role in the development of APF. By implementing the topological analysis to the downstream target of HAND2-AS1, it was found that only hsa-miRNA570-3p directly interacted with it. This also indicated that hsa-miRNA-570-3p may be the critical miRNA regulating APF.

APF has been shown to be closely related to inflammation and oxidative stress [35, 36]. Studies have reported that hsa-miRNA-570-3p is a novel regulator of inflammation [37]. It mainly participates in the occurrence and development of inflammation by regulating the expression of IL-6 and CXCL8, and these inflammatory factors have been confirmed to be closely associated with APF [38–40]. Moreover, a previous research reported the negative regulatory effect of has-miRNA-570-3p against MMP-9, which suggests this miRNA is closely related to the stability of the atherosclerotic plagues [37, 41].

The functionality of lncRNA HAND2-AS1 has been well characterized in several types of human cancers [42, 43]. Furthermore, studies have confirmed that lncRNA HAND2-AS1 is positively related to insulin-like growth factor I (IGF-1) [44]. And IGF-1 has also been proven to reduce lipid oxidation and foam cell formation by downregulating 12/15-LOX and ultimately reduce APF [45]. A previous study revealed that lncRNA HAND2-AS1 could regulate the expression of hypoxia-inducible factor-1*α* (HIF-1*α*) [46]. HIF-1*α* has also been confirmed to promote macrophage necroptosis by regulating miRNA-210 and miRNA-383 [47]. However, there is no evidence to point out the regulatory role of lncRNA HAND2-AS1 in APF.

As far as we know, this research constructed a ceRNA network containing lncRNA HAND2-AS1, hsa-miRNA-570-3p, and APF-related genes in APF. Furthermore, KEGG analysis has shown that the “regulation of platelet-derived growth factor receptor signaling pathway” may be the main pathway resulting in APF. It was previously reported that platelet-derived growth factor receptor (PDGFR) signaling pathway could regulate smooth muscle cell (SMC) migration and proliferation in the vascular wall, leading to increased predisposition to cardiovascular disease [48]. It has been reported that patients with APF exhibit higher PDGFR expression in clinical study [49]. In vivo and in vitro experiments have also confirmed that PDGFR can promote the development of APF [50]. Therefore, understanding the PDGFR signaling pathway in the development of APF is helpful to find novel treatments.

## 5. Conclusions

In this study, a global triple network including lncRNAs, miRNAs, and mRNAs was constructed based on the ceRNA hypothesis, and then, a regulatory network of APF was screened. lncRNA HAND2-AS1, hsa-miRNA-570-3p, DPP4, MYOCD, CNTN4, and CNTN1 were identified as the key genes. These findings complemented our understanding of APF and may provide potential diagnostic biomarkers or therapeutic targets in APF.

## Figures and Tables

**Table 1:** Nine DEMs screened from GSE43292.

Regulation	Gene symbol	logFC	*P* value
Upregulation	FABP4	2.454461	6.05*E*-08
	IBSP	1.794982	1.03*E*-08
	DPP4	1.610873	2.24*E*-07
Downregulation	chr4:174457998-174460620	−1.50436	1.39*E*-07
	FHL5	−1.56465	1.17*E*-08
	MYOCD	−1.62845	6.08*E*-08
	CASQ2	−1.66766	2.16*E*-08
	CNTN4	−1.79233	3.57*E*-09
	CNTN1	−1.91103	3.00*E*-08

**Table 2:** 39 DELs screened from GSE97210.

Regulation	Gene symbol	logFC	*P* value
Upregulation	PRDM16-DT	7.58	2.39*E*-10
	PART1	7.44	3.27*E*-11
	HAND2-AS1	6.83	7.03*E*-11
	LINC01252	6.33	1.4*E*-10
	PARD3-DT	6.04	1.71*E*-10
	LOC100507334	5.82	1.93*E*-10
	TBX2-AS1	5.69	1.18*E*-09
	SSTR5-AS1	5.48	7.59*E*-10
	PSORS1C3	5.37	4.16*E*-10
	LINC01139	5.08	2.41*E*-10
	SAMMSON	5.06	9.54*E*-10
	LOC100129455	4.96	3.86*E*-10
	LOC105369957	4.93	7.19*E*-10
	LOC105379447	4.88	4.86*E*-10
	LINC00165	4.86	4.01*E*-10
	SPANXA2-OT1	4.83	7.52*E*-10
	TMEM92-AS1	4.75	8.96*E*-10
	GATA6-AS1	4.69	1.25*E*-09
	KIF23-AS1	4.67	5.30*E*-10
	LINC02718	4.64	6.24*E*-10
	FSIP2-AS1	4.53	4.49*E*-10
	HOXC-AS3	4.50	5.78*E*-10
	CNN3-DT	4.12	5.36*E*-10
	LOC100506725	4.10	6.66*E*-10
	LOC105374029	3.98	8.05*E*-10
	LOC100507547	3.93	8.26*E*-10
	LOC100130992	3.87	1.24*E*-09
Downregulation	SUGCT-AS1	-4.60	4.10*E*-10
	LOC101927602	-4.71	4.78*E*-10
	VPS13A-AS1	-4.73	4.39*E*-10
	C1RL-AS1	-4.76	5.67*E*-10
	LOC105377323	-4.89	3.50*E*-10
	APOC1P1	-5.34	3.49*E*-10
	GAPLINC	-5.44	8.44*E*-10
	LINC00900	-5.84	2.27*E*-10
	L3MBTL4-AS1	-5.93	1.27*E*-10
	LINC01827	-6.27	8.47*E*-10
	CNIH3-AS2	-6.48	4.64*E*-10
	MMP2-AS1	-6.49	1.68*E*-10

**(a):** 

lncRNA	Degree	Closeness centrality	Betweenness centrality
HAND2-AS1	142	0.36317829457364337	0.20723471560902113
LINC00900	72	0.3457564575645757	0.10401877331726586
TBX2-AS1	12	0.20647862494490965	0.021243238559140364
GATA6-AS1	12	0.24657894736842106	0.019403039645996233
SPANXA2-OT1	6	0.22193273330175273	0.002378732808397449
SSTR5-AS1	6	0.2113216057735679	0.006399994526995594

**(b):** 

miRNA	Degree	Closeness centrality	Betweenness centrality
Hsa-miRNA-570-3p	5	0.41644444444444445	0.022132585913046563
Hsa-miRNA-466	5	0.38783112582781454	0.014465726762748462 6
Hsa-miRNA-4495	5	0.380275974025974	0.01051210577447764264

**(c):** 

mRNA	Degree	Closeness centrality	Betweenness centrality
CNTN1	289	0.4157054125998226	0.423378721015294453
MYOCD	226	0.3904166666666667	0.3305499570867205
CNTN4	177	0.3781275221953188	0.2358729852351261564
FHL5	108	0.3557327258921792	0.1310673691240780733
DPP4	101	0.3538519637462236	0.1366076949975910749
IBSP	60	0.3322695035460993	0.061990908379872794
CASQ2	56	0.33584229390681003	0.07356388148731316
FABP4	26	0.30206318504190843	0.024157518050064344

## Data Availability

All of the data was obtained from the GEO database (http://www.ncbi.nlm.nih.gov/geo). The lncRNA-miRNA and miRNA-mRNA interaction data were obtained from the lncRNASNP database (http://bioinfo.life.hust.edu.cn/lncRNASNP/) and starBase V2.0 database (http://starbase.sysu.edu.cn/).

## Abbreviations

AS: Atherosclerosis
lncRNAs: Long non-coding RNAs
APF: Atherosclerotic plaque formation
DEGs: Differentially expressed genes
miRNAs: MicroRNAs
ceRNA: Competitive endogenous RNA
FDR: False discovery rate
GO: Gene ontology
DELs: Differentially expressed lncRNAs
DEMs: Differentially expressed mRNAs
DEMis: Differentially expressed miRNAs.
